# Supplementary material for: An Integrative Study on Asphondylia spp. (Diptera: Cecidomyiidae), Causing Flower Galls on Lamiaceae, with Description, Phenology, and Associated Fungi of Two New Species
Source: Insects. 2021 Oct 21;12(11):958. doi: 10.3390/insects12110958 (PMC8619354; doi:10.3390/insects12110958)
Supplement: Supplementary file 1 [file insects-12-00958-s001.zip › insects-1403973-supplementary.pdf]

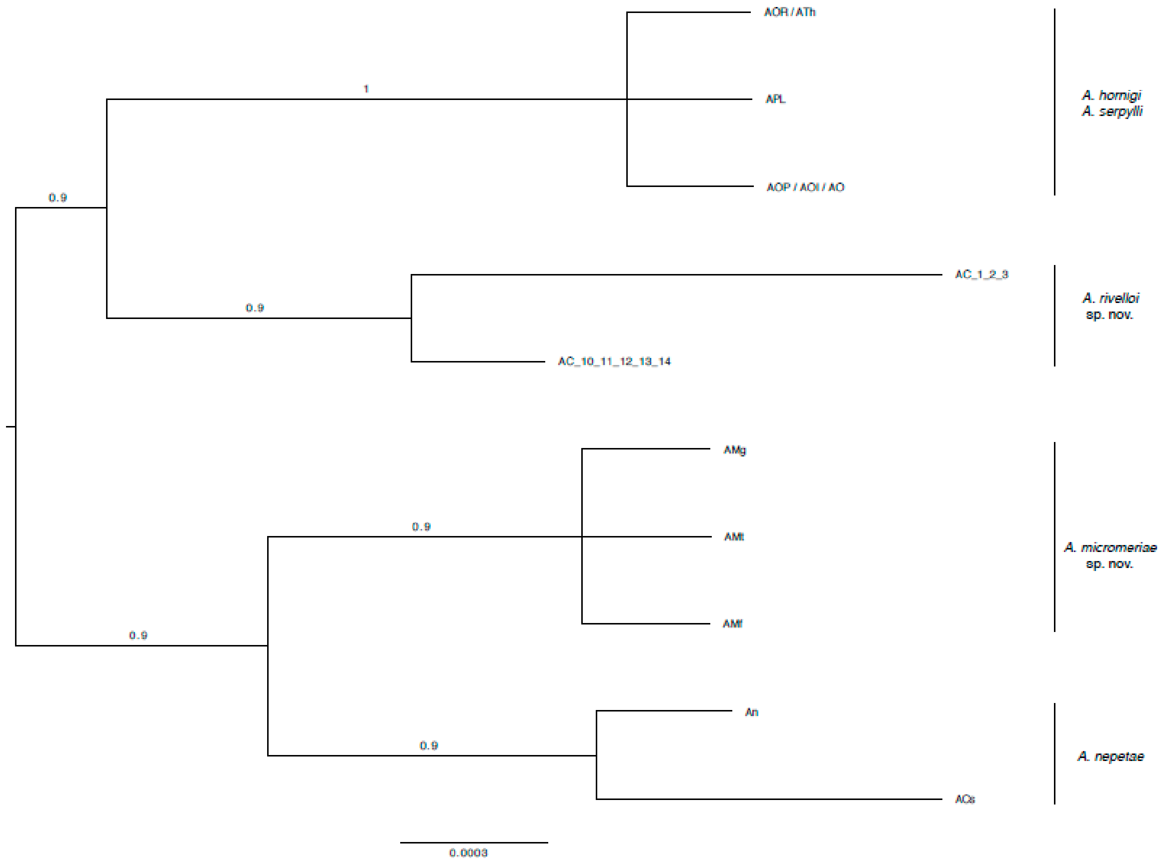

Figure S1: Bayesian majority rule consensus tree based on combined nuclear markers (ITS2+28S-D2). Posterior probabilities ≥ 0.9 are shown above branches. The tree was rooted using the midpoint-rooted tree option.

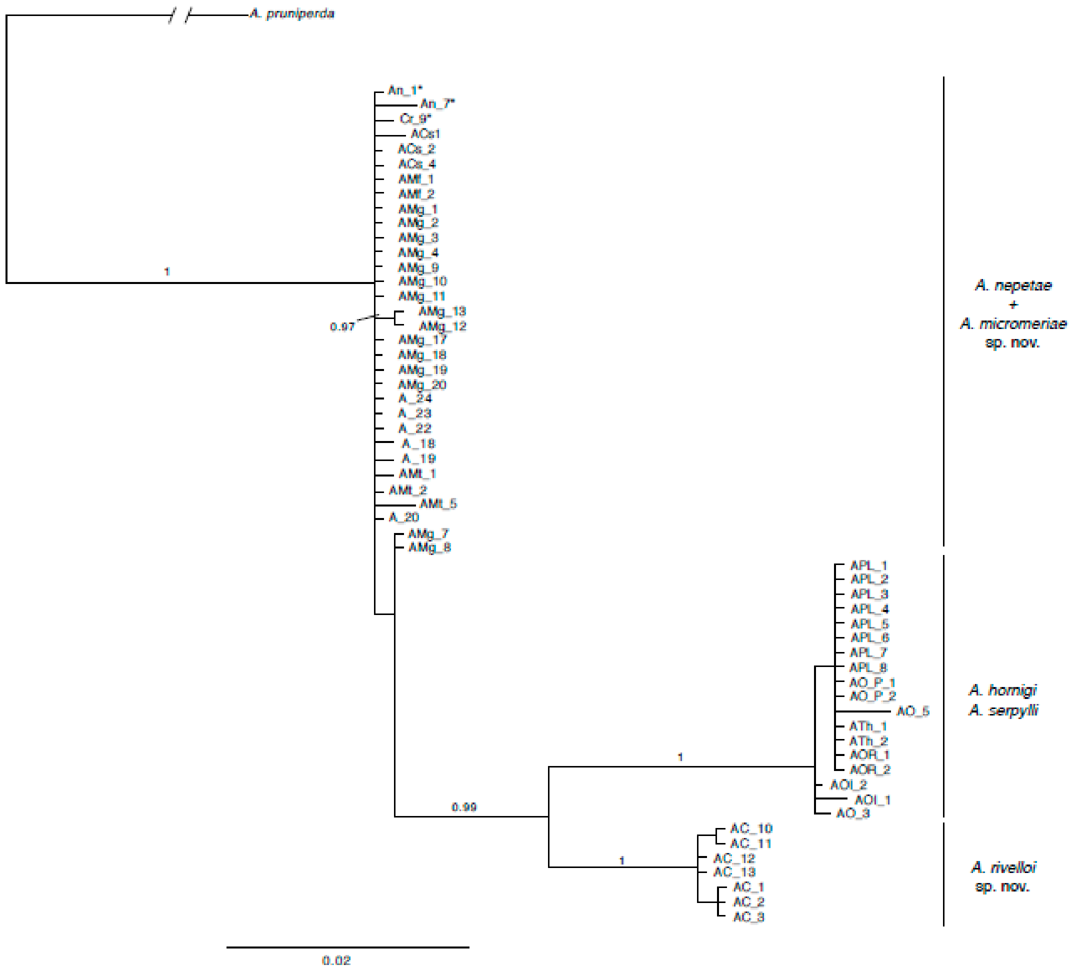

Figure S2: Bayesian majority rule consensus tree based on COI alignments of *Asphondylia* specimens of the present work and sequences (*) from [4]. Posterior probabilities ≥ 0.9 are shown above branches.

Table S1. Differences in nuclear genes ITS2+28S-D2 among the studied *Asphondylia* species.

Species	Sites/bp							
	70	122	183	307-336	374	410	415	888
A. nepetae	A	T	T	-	G	C/T	G	A
A. rivelloi sp. nov.	G	T	T	ATACATTAAATAAATACCAAGAAATATTAT	-	C/T	A/G	A
A. micromeriae sp. nov.	A	T	T	ATACATTAAATAAATACCAAGAAATATTAT	G	T	G	G
A. hornigi *A. serpylli*	A	A	G	ATACATTAAATAAATACCAAGAAATATTAT	-	T	G	A

Table S2. Length/maximum width ratios of *A. nepetae* and *A. micromeriae* flagellomeres.

Antennal flagellomere	Species	
	A. nepetae	*A. micromeriae*
F1	6.14	4.51
F2	4.12	3.6
F3	4.00	3.18
F4	3.75	3.14
F5	3.75	3.19
F6	3.50	3.10
F7	4.00	2.88
F8	2.62	2.07
F9	2.25	1.64
F10	1.30	1.00
F11	1.00	0.94
F12	1.00	0.83
